# Research advances in microneme protein 3 of *Toxoplasma gondii*

**DOI:** 10.1186/s13071-015-1001-4

**Published:** 2015-07-22

**Authors:** Yanhua Wang, Hong Yin

**Affiliations:** State Key Laboratory of Veterinary Etiological Biology, Lanzhou Veterinary Research Institute, Chinese Academy of Agricultural Sciences, Lanzhou, 730046 China

**Keywords:** Toxoplasmosis, Microneme protein 3, Function, Diagnostic agents and vaccines

## Abstract

*Toxoplasma gondii* (*T. gondii*) is an obligate intracellular protozoan parasite. It has extensive host populations and is prevalent globally; *T. gondii* infection can cause a zoonotic parasitic disease. Microneme protein 3 (MIC3) is a secreted protein that is expressed in all stages of the *T. gondii* life cycle. It has strong immunoreactivity and plays an important role in the recognition, adhesion and invasion of host cells by *T. gondii*. This article reviews the molecular structure of MIC3, its role in the invasion of host cells by parasites, its relationship with parasite virulence, and its induction of immune protection to lay a solid foundation for an in-depth study of potential diagnostic agents and vaccines for preventing toxoplasmosis.

## Review

### Background

**Fig. 1:**
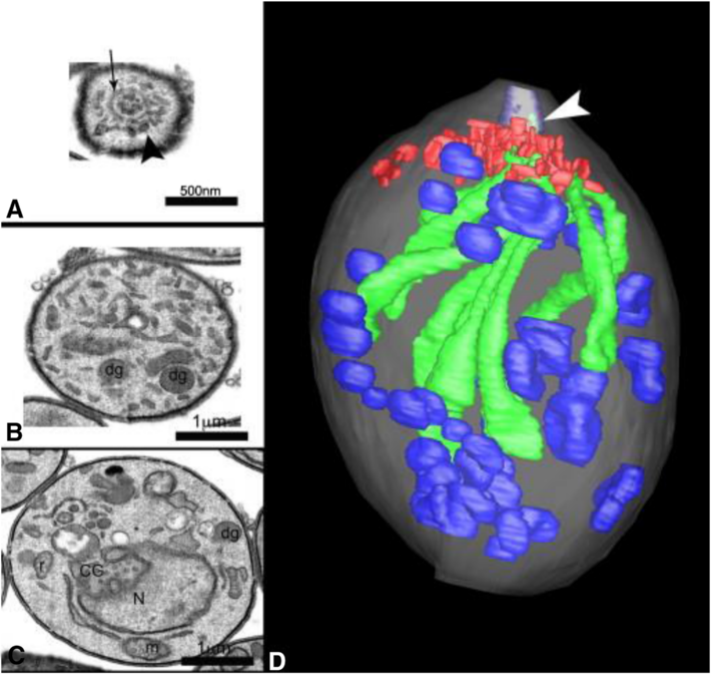
3D reconstruction of an extracellular *T. gondii* tachyzoite. Reprinted from [1], Copyright ©2011, with permission from Elsevier. **a**–**d** Transversal slices of a parasite used for reconstruction from the apical (**a**), **b** to the middle **c** and posterior portions of the cell body, by FIB-SEM dual beam. **a** Face view of the polar ring (arrow) with micronemes (arrowhead) around it and rhoptry necks inside. **b** Between the polar ring and the nucleus, a rhoptry (r) and a dense granule (dg) can be identified. **c** At the upper level of the nucleus (N) elements from the Golgi complex (GC) and mitochondrial profiles (m) are seen. **d** Rendered model. The plasma membrane is transparent white. Inside the parasite several dense granules (blue) are scattered in the cytoplasm, 10 rhoptries (green) and many micronemes (red) are seen around the conoid (arrow). The neck of one rhoptry is seen inside the conoid (white arrow) [1]

*Toxoplasma gondii* (*T. gondii*) is an obligate intracellular protozoan parasite (Fig. 1) [1]. It has extensive host populations, is prevalent globally, and can cause a zoonotic parasitic disease [2, 3]. As an opportunistic infection factor, *T. gondii* can cause death in patients with impaired immune function or immune suppression, such as acquired immunodeficiency syndrome (AIDS) patients, organ transplantation patients, and malignant tumor patients. Toxoplasmosis is also an important biological factor influencing human prenatal and postnatal care because if a pregnant woman is infected with *T. gondii*, maternal-fetal vertical transmission may occur and result in miscarriage, stillbirth or congenital defects or deformities (malformation, retardation, etc.) in fetus [4]. The major hazards after *T. gondii* infection in animals is the treatment cost after outbreak of toxoplasmosis and the direct loss caused by animal death; another hazard is the indirect loss caused by long-term parasite hosting to become a potential infection source and cause reproductive disorders [5].

**Fig. 2:**
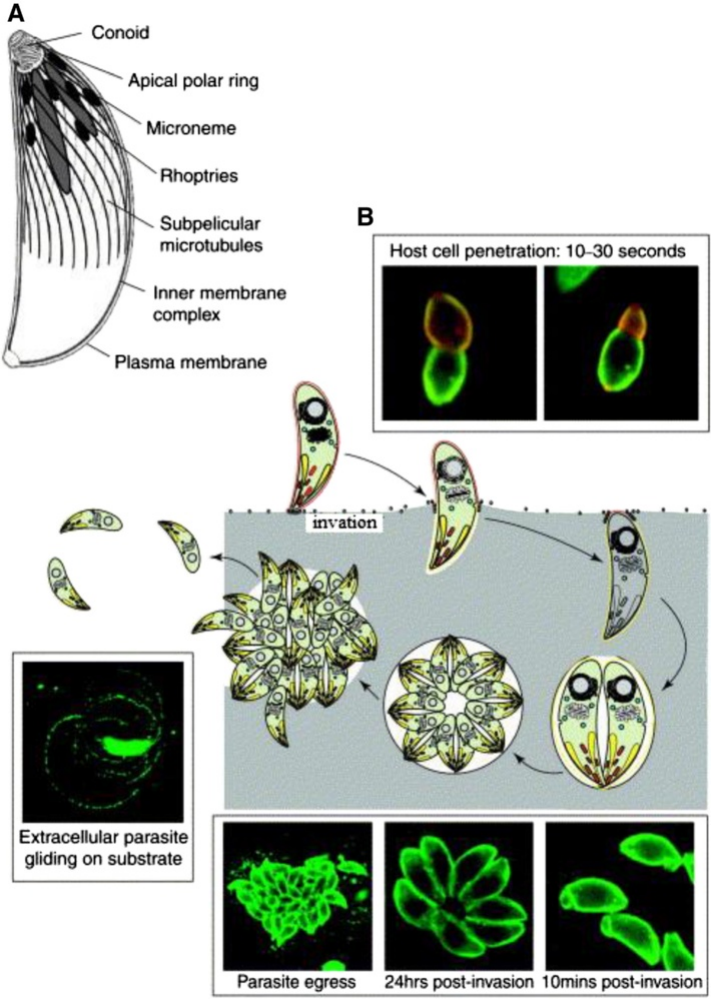
Host cell invasion by *T. gondii*. Reprinted from [11], Copyright © 2004, with permission from Elsevier. **a** Schematic representation of *T. gondii* tachyzoite and the subcellular structures involved in gliding motility and host cell invasion. **b** Cycle of host cell invasion and egress by *T. gondii*. This multi-step process includes the attachment to host cells, the discharge by the micronemes (red), the discharge by the rhoptries (yellow), the formation and sealing of the parasitophorous vacuole, intracellular parasite replication, lysis of the PVM and parasite egress [11]

*T. gondii* is highly evolved to form a complex, well-coordinated system. It regulates the secretion pathway to supplement proteins for immune regulation, gliding motility, nutritional requirements in the intracellular survival and replication process after invasion into hosts, and the final release after invasion [6]. Continuous observation of the process of invasion into hosts by *T. gondii* by electron microscopy showed that the invasion of host cells by *T. gondii* is not similar to that of other intracellular pathogens that require making full use of the phagocytosis function of host cells for invasion. *T. gondii* utilize an active invasion process, relying on its energy and the “gliding motility” produced by its actin-myosin system (Fig. 2) [7–11]. The completion of this process depends on a series of functions by different organelles at specific times, including microneme proteins (MIC), rhoptry proteins (ROP), and dense granule antigens (GRA) [12]. Among these, MICs secreted by the microneme play a basic role in the recognition, adhesion, and invasion of parasites into host cells during the invasion process. At the early stage of contact between parasites and host cells, MICs are first secreted from the apex of tachyzoites and facilitate adhesion through the recognition of receptors on the cell membrane of hosts; thus, they play an important role in the early stage of the invasion of host cells by parasites [13]. Currently, there are at least 19 types of known MICs (Table 1) [14–34], including MIC1-MIC12, AMA1, M2AP, SUB1, ROM1, SPATR, PLP1, and TLN4, of which 10 types (TgMIC1-4, TgMIC6-9, TgMIC12, and SPATR) contain different adhesion domains similar to the adhesion molecules in eukaryotic cells (integrin-like domain, thrombospondin type-1 repeat (TSR), epidermal-growth factor (EGF)-like domain, chitin binding-like (CBL) domain, and the Apple domain). These proteins play a role in the process of recognition and adhesion of host cells by parasites [6]. MIC3 can bind to the receptors of a variety of host cells through its ligand structural domain; thus, it is closely associated with the invasion of host cells by parasites and the virulence of the infection. In addition, MIC3 is expressed in the tachyzoite, bradyzoite, and sporozoite stages and has excellent immune effects. Therefore, MIC3 has received extensive attention. This article reviews the structure and function of MIC3, its function in the invasion of host cells by parasites, its association with the virulence of parasites, and its induced immune protection.

**Table 1:** Properties of *Toxoplasma* microneme proteins

MICs	MW(kDa)^a^	Domains/homologies	Major putative functions	References
MIC1	49	2 TSRs	Transport/folding of MIC4 and MIC6	[14, 15]
			Binding to host cells	
MIC2	83	1 Integrin, 5 TSRs, and 1 TM	Transport M2AP and adhesion	[16, 17]
MIC3	38(90 dimer)	1 CBL and 5 EGF	Adhesion	[14, 18]
MIC4	63	6 Apples	Adhesion	[19, 20]
MIC5	20	Parvulin-like PPIase motif	Suppressing TgSUB1 activity	[21]
MIC6	37	3 EGF-like	Escorter	[20]
MIC7	36	5 EGF-like and 1 TM	unkown	[13]
MIC8	75	1 CBL, 10 EGF-like, and 1 TM	Escorter	[13]
MIC9	32	3 EGF and 1 TM	unkown	[13]
MIC10	23		unkown	[22]
MIC11	22		unkown	[23]
MIC12	234	31 EGF, 4 TSRs, and 1 TM	unkown	[24]
AMA1	60	Cysteine rich	Inhibiting secretion of the rhoptries	[25]
M2AP	35	Beta and coil	Transport/folding of MIC2	[26, 27]
SUB1	85	Subtilase and GPI	Proteolysis	[28]
ROM1	28	Rhomboid	Proteolysis	[29, 30]
SPATR	47.5^b^	1 thrombomodulin EGF, 2 TSRs	Trafficking of the protein	[31]
PLP1	124		Cytolysis and parasite egress	[32, 33]
TLN4	256		unkown	[34]

^a^Based on the complete open reading frame including signal sequence or GPI anchor signal; ^b^Predicted molecular weight (MW) after removal of the signal peptide

Abbreviation: *TM* transmembrane

### Molecular characteristics of MIC3

**Fig. 3:**
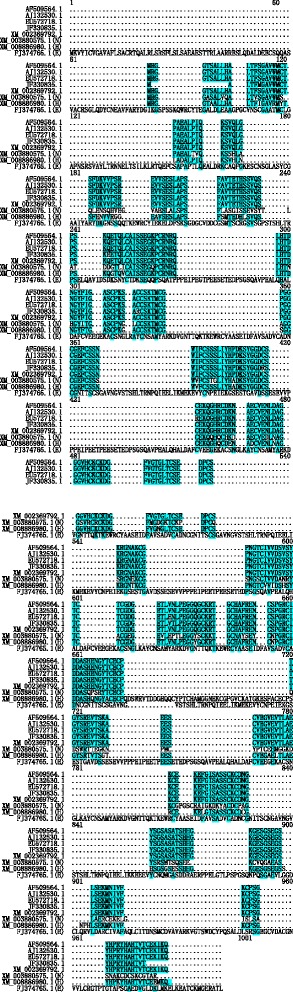
Amino acid sequence alignment of MIC3 from apicomplexan parasites. *Toxoplasma* MIC3 (AF509564.1: RH.SSI strain; AJ132530.1: RH.ERP strain; EU572718.1 GJS strain; JF330835.1 RH strain, and XM_002369792.1: ME49 strain), *Neospora caninum* (XM_003880575.1: Liverpool strain), *Hamondia Hammond* MIC3 (XM_008886980.1: H.H.34 strain), and *Eimeria tenella* (FJ374765.1: Houghton strain). Amino acid sequence alignment was generated by MegAlign. Identical amino acid residues are colored in blue

**Fig. 4:**
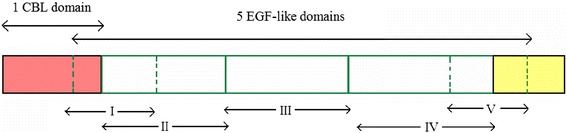
Schematic drawing of the MIC3 constructs. EGF domains II, III, and IV are tandemly repeated. EGF domains I and V overlap with other domains

The length of the mic3 gene sequence is 1,080 bp; the sequence encodes 359 amino acids and a protein with a molecular weight of 38 kD. The mic3 gene is a single copy gene; it does not have any introns, and there is not a eukaryote-specific TATA or CCAAT promoter sequence at the 5’-end non-coding region. The complete open reading frame defined by the first ATG codon encodes 359 amino acids, which contain 34 cysteine residues. There is a short hydrophobic signal peptide sequence at the N terminus, and there is no transmembrane protein domain. There is 1 potential glycosylation site and 12 phosphorylation sites. Amino acid sequence identities of MIC3 among different *Toxoplasma* strains are very high, more than 98.3 % (Fig. 3). In contrast, large sequence differences exist among different apicomplexan parasites (Fig. 3). MIC3 structure contains 5 partially overlapped EGF-like domains, of which 3 domains form a tandem repeat and the other 2 domains are overlapped with the former 3 domains, as well as a CBL domain (Fig. 4). The CBL domain may be involved in protein-protein and protein-carbohydrate interactions [18]. Cèrède *et al.* reported that the MIC3 of *T. gondii* tachyzoites is a 38-kDa (monomer) end product from the proteolytic digestion of a 40-kDa precursor, which is then folded into a 90-kDa dimer [35]. These specific structures are closely associated with the function of MIC3. Amino acid mutagenesis experiments confirmed that the formation of MIC3 dimers did not rely on disulfide bonds between peptides and may be instead associated with the interaction between proteins. The dimer is necessary for the adhesion function. EGF-like domains are present in many proteins; they are distributed in the extracellular region of membrane-binding proteins or secreted proteins through a tandem repeat form. Their function is still not completely clear. To investigate whether MIC3 interacted with the EGF-like receptors of host cells through the EGF-like domain, adhesion experiments were conducted between MIC3 and cells. The experimental results showed that regardless of whether cells had EGF-like receptors, there were no differences among the adhesion results. This finding indicates that the EGF-like domain was either not involved in adhesion between MIC3 and cells or that the EGF-like domain did not interact with the EGF-like receptors in host cells. Through the expression of different MIC3 fragments on the surface of host cells, it was elucidated that the CBL domain was necessary for the adhesion characteristics of MIC3. In contrast, the EGF-like domain could not promote adhesion of parasite MIC3 to host cells but may enable the accurate presentation of the adhesion structure from the structure formation aspect.

### The MIC3 complex of *T. gondii*

**Fig. 5:**
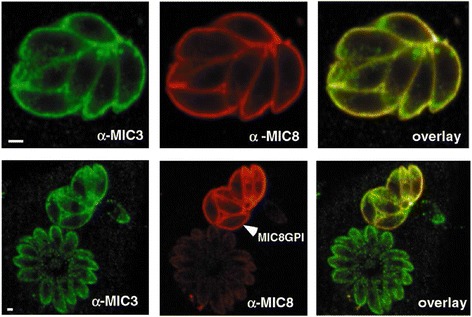
MIC8 serves as escorter for the non-membrane adhesin TgMIC3. Reprinted from [13], Copyright © 2002, with permission from Elsevier. Double IFA analysis by confocal microscopy of parasites transiently transfected with pTMIC8GPI. TgMIC8 covalently linked to a GPI anchor localized perfectly at the plasma membrane of the parasites. TgMIC3 redistributed to the plasma membrane in the transiently transfected parasites, while the protein is perfectly sorted to the micronemes in a vacuole containing non-transfected parasites [13]

**Fig. 6:**
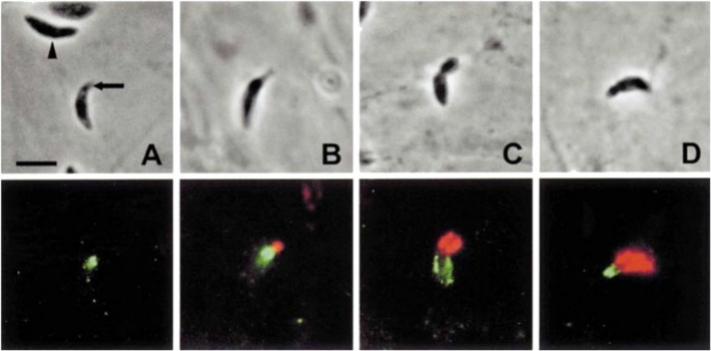
Immunofluorescence localization of MIC3 (green) and ROP1 (red) during invasion of HFF cells. Reprinted from [18], Copyright © 2000, with permission from John Wiley and Sons. **a**, **b**, **c** and **d** are successive steps of invasion [18]

During the process of secretion, transport, and release, MICs interact with other MICs to function in a complex form. Currently, the known MIC complexes include the MIC1/4/6 complex, MIC3/8 complex, MIC2/M2AP complex and AMA1/RON2/RON4/RON5/RON8. At least one protein in the complex contains a transmembrane domain and a cytoplasmic tail. The cytoplasmic tail contains a sorting signal. The sorting signal is necessary for transporting the complex from the endoplasmic reticulum (ER) to the relevant locations in the micronemes; thus, this protein is defined as an “escorter” [13, 20] and is called a transmembrane protein. MIC8 is the escorter of the MIC3/8 complex (Fig. 5) [13]. MIC3 reaches micronemes under the escort of its escorter, MIC8. It is discharged outside parasites during the process of invasion of the host cell by parasites and is then re-localized on the surface of the host cells (Fig. 6) [18]. Proteins that interact with escorters are soluble proteins. The normal execution of transmembrane protein functions also depends on its interacting soluble proteins. Only following an interaction between soluble proteins and transmembrane proteins can they be correctly folded to form a complex with biological functions; otherwise, the function of the complex will be damaged. For example, following disruption of the mic1 gene of *T. gondii*, its partner proteins, MIC6 and MIC4, remained in the Golgi apparatus [20]; when the m2ap gene of *T. gondii* was knocked out, MIC2 was retained and accumulated in the Golgi apparatus, thus causing a significant decrease in MIC expression levels [27]. Although the interactions between proteins are very important, there are some exceptions. For example, after the mic3 gene of *T gondii* was knocked out, the transport of MIC8 to the micronemes and secretion of MIC8 in the micronemes were not affected [14]. These stable complexes better explain how soluble proteins are sorted after regulation and secretion and how soluble adhesins assist the invasion process [36, 37]. However, whether the functions of these complexes in the motility and cell invasion processes of *T. gondii* are repeated or redundant are still unknown [14, 25].

### Regulatory mechanisms of MIC secretion

Micronemes are secretion organs that are located at the apical ends of parasites. MICs are synthesized at the rough ER, are transported to micronemes through the Golgi apparatus and are secreted outside the body. It is generally accepted that the secretion of *T. gondii* MICs is only regulated by the intracellular calcium ion levels in parasites [38]. Under normal conditions, *T. gondii* tachyzoites secret MICs at a very low level; when the intracellular calcium ion level increases, the secretion of MICs can increase 10-100-fold [39]. The imbalance of calcium ions will induce the deregulation of MIC secretion. Some reagents that can neutralize intracellular calcium ions can strongly inhibit MIC secretion by micronemes. The regulation of MIC secretion by calcium ions in parasites does not require the participation of exogenous calcium ions [40, 41]. Additionally, through the research of microneme secretion based on levels of MIC5, Carey *et al.* extrapolated that there could be two types of micronemes: one that discharges rapidly in response to elevated intracellular calcium, and one that discharges constitutively [42]. Further larger trials are needed to determine whether two types of micronemes exist.

### MIC3 protein folding and hydrolysis

Newly synthesized proteins require structural modification at the ER. Misfolded or incompletely folded oligomeric complexes will be retained at the ER and will finally be degraded. There are some molecular chaperones and folding enzymes at the ER; they can assist the correct folding and assembly of proteins and can provide anchor points for retained immature proteins [43]. The retention functions of ER rely on different folding sensors; the N-glycans on the surface of misfolded proteins can be recognized by calreticulin and calnexin. The exposed cysteine residue plays a role in thiol-mediated protein retention; under this condition, thiol oxidoreductase on the ER membrane is associated with the retention of unassembled proteins. MIC3, MIC4, and MIC6 are rich in cysteine residues [35]. When the early peptides are transported to the ER, cysteine residues form disulfide conjugates to perform correct folding. Incorrectly folded proteins may form aggregates and cannot be correctly transported.

The hydrolysis processing of MICs is very common and is necessary for the smooth completion of the invasion process, which mainly occurs in 2 stages. The first stage occurs at the secretory pathway, that is, transportation to the micronemes through the Golgi apparatus after synthesis by the rough ER. The hydrolysis process at this stage is associated with the formation of specific complexes, correct targeting of complexes to organelles, and masking of enzyme active sites. MIC3, MIC5, MIC6, and M2AP are actively involved in this stage [20, 44]. The second stage occurs after extracellular secretion and discharge during the process of the invasion of host cells by *T. gondii*. The processing at the second stage is helpful for the completion of complex separation, host cell adhesion, and invasion of parasites. For example, the protein processing of MIC2, MIC4, MIC6, and MIC8 induce the rapid release of MICs on the cell surface, which is a key step for completion of the cell invasion. If processing is lacking, secreted MICs can establish persistent, stable bridges on the surface of host cells and parasites, thus interfering with the newly formed correct closure of parasitic vesicles and inhibiting the proliferation of parasites in vacuoles. Studies showed that unprocessed MICs increased adhesion to host cells; however, the invaded parasites decreased. Many MICs are produced as a pre-protein form and go through proteolytic processing during the secretion pathway and transport process. When crossing the Golgi apparatus, the first EGF-like domain of MIC6 is removed [13]; the function of this process is unknown, but this step does not affect the microneme targeting ability of the interaction between MIC6 and MIC1 [20]. MIC3 is also initially synthesized as a 40-kDa dimeric precursor, which then becomes a 38-kDa product through proteolysis before reaching the micronemes [45]. The removal of the leader peptides of MICs is necessary for interaction with the host cells [46]. The enzymes that hydrolyze *T. gondii* MICs have not been completely clarified.

### The function of MIC3 in the process of invasion of the host cells by parasites

*T. gondii* can invade almost all nucleated cells, indicating that there are multiple “receptor-ligand” interactions between parasites and host cells. Although the mechanisms underlying the invasion of host cells by *T. gondii* have not been completely clarified, current studies indicate that *T. gondii* actively invades cells, unlike the endocytosis processes of infection used by bacteria and viruses. When *T. gondii* contacts host cells, parasites first push the conoid outside the body, and then, micronemes discharge a large amount of MICs through the conoid. MICs interact with corresponding receptors on the host cell membrane and are distributed on the surface of parasites. The front end of parasites, which interacts with cells, forms a moving junction [47]; next, the actin-myosin system exerts its function. At the moving junction of parasites, MICs move from the front end of parasites to the rear end of parasites through the force produced by actin proteins and are finally hydrolyzed; parasites invade cells through the moving junctions [8, 11, 35, 47, 48]. Therefore, MICs, including MIC3, play a bridging role between parasites and host cells.

It has been shown that mic1 knockout alone affected the invasion of HFF cells by *T. gondii* and that mic3 knockout alone did not affect the invasion; the results of a mic3/mic1 double knockout did not show significant differences from the mic1 knockout. These results indicate that these 2 proteins did not have synergistic effects on *T. gondii* invasion [49]. These results contradict those showing that MIC3 was secreted outside cells during the invasion process of *T. gondii* and adhered to a variety of cells, including fibroblasts, macrophages, and epithelial cells. Cérède *et al.* [14] proposed that MICs may contain a large number of components and that different components could recognize the same receptor. For example, during the infection of HFF cells after mic3 knockout, other MICs would interact with the cell surface receptors; however, MIC3 may be indispensable for the infection of other cell types. In addition, during the invasion process, parasites may have alternative invasion routes. Furthermore, *in vitro* culture and natural infection methods may display differences; in *in vitro* culture, the disappearance of one gene function may be compensated for by other MICs or may up-reregulate the secretion of other MICs to allow for normal invasion; however, this gene may be very important in natural infection conditions.

### The association between MIC3 and the virulence of parasites

MICs contain a series of conserved adhesion domains, and some domains have been confirmed to be associated with parasite invasion [14]. Following infection of HFF cells after the *T. gondii* mic3 gene was knocked out, the adhesion characteristics were not affected, but the parasite virulence significantly reduced. *T. gondii* with gene disruption of mic1 or mic3 were inoculated into mice, and all of the inoculated mice died after 9–22 days of inoculation; mice in the control group without gene knockout all died within 7–10 days. These results indicated that after *T. gondii* mic1 and mic3 were separately knocked out, the virulence of *T. gondii* only decreased slightly. *T. gondii* with both the mic1 and mic3 genes knocked out were inoculated into mice, and the results showed that only 1 mouse among the 10 mice died and that the death time of mouse was delayed by 10 days compared to that of the control group. In addition, the dose was adjusted and inoculated into mice to cause the death of all mice on day 9; comparison of lethal doses with the control group showed that the inoculation amount of the double gene knockout *T gondii* was 2 × 10^3^ parasite/mouse and that the dose in the control group was lower than 20 parasite/mouse. These results indicate that knockout of the mic1 and mic3 genes together significantly reduced the parasite virulence and confirmed that MIC1 and MIC3 had synergistic effects on the parasite virulence [14].

MIC3 has a very strong affinity for host cells, which is closely associated with its CBL domain [35]. The common sequences of CBL domain contain 8 disulfide bond-linked cysteine residues and several highly conserved aromatic residues. BHK-21 cells were transfected with the recombinant MIC3 plasmid, and the location of the expression product was detected. The results showed that the expression product of the recombinant MIC3 with CBL domain knockout was in the supernatant, whereas the expression product of the recombinant MIC3 without CBL domain knockout was on the cell surface. These results indicate that the CBL domain of *T. gondii* MIC3 is very important for cell adhesion.

In CBL domain, Cysteines determine the protein conformation, and aromatic residues react with N-acetylglucosamine. Transfection of BHK-21 cells with the recombinant MIC3 plasmid in which all the cysteine residues in CBL domain were replaced by glycine residues resulted in insufficient secretion and accumulation of the expression product, indicating that cysteine residues determine the protein conformation. In the aromatic residue replacement experiment, the F121A and Y141A mutants influenced the protein expression in BHK-21 cells; however, the adhesion characteristics of these two residues could not be confirmed. The transfection experiments of the other recombinants (Y96A, F97A, P103A, W126A, F128A, and Y135A) proved that only the expression products of the W126A and F128A mutants were in the supernatant and did not bind to the surface of transfected BHK-21 cells, indicating that the replacement of W126 or F128 alone resulted in the complete loss of adhesion function and that they were closely associated with the interaction with cell surface receptors and adhesion. The replacement of tryptophan at position 126 with glycine (W126A) and the replacement of phenylalanine at position 128 with glycine (F128A) in the CBL domain of MIC3 caused a significant reduction of virulence of the mutant strains. These studies indicated that the MIC3 CBL domain that is associated with adhesion is very important for the virulence of parasites [14].

### Induction of immune protection by MIC3

MIC3 plays important roles in the invasion process of *T. gondii*; it has very strong immunogenicity and can stimulate the production of corresponding humoral immunity and cellular immunity in the body. Therefore, in studies of vaccines against *T. gondii* infection, MICs have received much attention. Vaccines based on MIC3 include live attenuated vaccines, subunit vaccines, and DNA vaccines.

### Gene deletion attenuated vaccine based on MIC3 of *T. gondii*

Some scholars have performed studies on live attenuated vaccines by knocking out double virulence genes of parasites. A mutant strain of *T. gondii* (RH strain) lacking the mic1 and mic3 genes (Mic1-3KO) tachyzoites to immunize mice and that showed mice could produce a strong protective function [14, 49]; not only could the mother defend against *T. gondii* infection but also vertical transmission could be effectively prevented [49]. Of note, the MIC1-3KO attenuated strain induced good immune effects in murine models and also had ideal immune effects in large animal models. Subcutaneously immunized ewes with MIC1-3KO tachyzoites showed that IgG antibodies occurred in serum samples. Ewes at mid-gestation were challenged with the PRU strain of *T. gondii*, and the results showed that pregnant ewes in the vaccinated group only had a slight febrile response, whereas unvaccinated ewes produced a more severe, characteristic febrile response of a longer duration; all unvaccinated ewes aborted. The survival rates of newborn lambs in the vaccinated group were 62 %, 91 %, and 64 %, and there were no clinical signs of infection. A dose of 10^5^ MIC1-3KO tachyzoites was sufficient to induce protective immune responses and had very good preventive function against toxoplasmosis-induced abortion in ewes [50]. At present the strain MIC1-3KO is the main product of a French innovative company (VitamFero SA) and it is called "Toxo KO" vaccine. "Toxo KO" vaccine represents a new generation of live and attenuated vaccines obtained through the deletion of 2 genes of virulence, and the risk of reversion to virulence is totally eliminated (http://www.vitamfero.com/en/technology.html). "Toxo KO" vaccine will play an important role in preventing ovine and feline toxoplasmosis.

### Subunit vaccines based on MIC3 of *T. gondii*

Subunit vaccines are vaccines developed using DNA recombinant technology to introduce antigenic genes of pathogens into prokaryotic or eukaryotic expression systems to express the corresponding antigenic proteins; these vaccines have the advantages of easy preparation of antigenic proteins and high yields. Nie *et al.* [51] vaccinated BALB/c mice with recombinant pseudorabies virus (PRV) expressing MIC3 (rPRV-MIC3), and the results showed that rPRV-MIC3 could induce significant humoral and Th1 cellular immune responses. After being challenged by the *T. gondii* RH strain at a lethal dose (100 tachyzoites), the survival rate of immunized mice was 50 %, and no protection was found in control groups. These results indicated that rMIC3 had better immunogenicity and could produce a certain protective immunity, which provided reliable bases for the development of MIC3 subunit vaccines for *T. gondii*. However, current subunit vaccines still have issues such as insufficient immunogenicity and protective function and too short of a persistent time of the immune responses.

### Nucleic acid vaccines based on MIC3 of *T. gondii*

*T. gondii* belongs to strictly intracellular parasitic protozoa. In induced immune responses in the body, cellular immunity is more important than humoral immunity; especially in the acute infection phase, cellular immunity usually plays a decisive role. DNA vaccines express corresponding antigenic proteins in host cells and comprehensively induce specific immune responses in the body. Compared to other vaccines, DNA vaccines have the potential advantages of safety, stability, convenience, high efficiency, and persistent immunity. Ismael *et al.* used the pMIC3i plasmid encoding the immature MIC3 protein to intramuscularly inoculate CBA/J mice. Immunized mice all produced high titers of IgG2a type anti-MIC3 immunoglobulin as well as IFN-γ and IL-2. Inoculation of mice with the pGM-CSF plasmid encoding the granulocyte-macrophage colony-stimulating factor gene and the pMIC3i plasmid together showed that immunized mice produced stronger humoral immune responses. Oral administration of cysts of the 76 K strain of *T. gondii* into immunized mice showed that the number of cysts detected in the brain was significantly lower than that in control mice [52]. Fang *et al.* [53] used the “suicidal” pSCA1 vector to construct a *T. gondii* suicidal DNA vaccine pSCA/MIC3. As conventional DNA vaccine pcDNA/MIC3, suicidal DNA vaccine pSCA/MIC3 also provides favourable efficacy. Meantime, based on the alphavirus replicon, suicidal DNA vaccines leads eventually to apoptosis of the transfected cells, which is particularly important in alleviating the concerns of potential integration and cell transformation generated by the use of conventional DNA vaccines, so it could improve safety of the conventional DNA vector. In this case, the design of vaccines against *T. gondii* based on suicidal DNA approach is feasible and effective. CD4 and CD8 T cells are the major effectors of the MIC3 DNA vaccine-induced protection [54, 55], both Lectin-like and EGF-like domains of MIC3 conferred protection [54]. Of note, the MIC3 DNA vaccine induced good immune effects in large animal models (sheep) [56].

Although monovalent DNA vaccines can induce a very strong immune reaction in the body against *T. gondii* infection, the preventive function is not comprehensive, and the immune effect is not ideal [57]. Hence, in the course of researching *T. gondii* vaccines, a consensus to develop multivalent vaccines that contain a combination of several antigens and target different developmental stages has been reached with the aim of overcoming the drawback of the monovalent candidate vaccines [58]. Qu *et al.* [59] immunized mice with the constructed composite DNA vaccine ZJ111/PSAG1-MIC3, and the results showed that mice could reduce stronger humoral and Th1-type cellular immunity than those in the control group (pSAG1, pMIC3, and pcDNA3.1). After being challenged by the *T. gondii* RH strain at a lethal dose for 12 days, the survival rate of mice was still above 20 %, whereas mice in the control group all died within 5–6 days. Therefore, compared to monovalent DNA vaccines, multivalent composite DNA vaccines can produce better protective immunity. Some other studies also support the view that the introduction of multiantigenic DNA vaccine is more powerful and efficient than single-gene vaccine, and deserves further evaluation and development [60, 61]. The multiantigenic DNA immunization might be an important approach to achieve an effective vaccine against *T. gondii*.

Cytokines are excellent adjuvants and can significantly enhance the immune effects of gene vaccines. IFN-γ is an immune upregulation factor that plays a leading role. During the process of resisting *T. gondii* infection by immune cell, IFN-γ plays an important immune regulation role [62]. The pcMIC3 eukaryotic expression plasmid encoding the *T. gondii* MIC3 and the pcIFN-γ eukaryotic expression plasmid encoding IFN-γ were mixed together at 100 μg each and were then intramuscularly injected into BALB/c mice 3 times at intervals of 2 weeks. The results showed that mice could effectively defend the challenge of a small dose of a strong virulent strain of *T. gondii*, indicating that the recombinant plasmids encoding the *T. gondii* MIC3 and IFN-γ had excellent immunogenicity to induce strong humoral immunity and cellular immunity [63]. Collectively, these results demonstrated that MIC3 is considered a very promising candidate molecule for the development of vaccines against toxoplasmosis.

Taken together, studies have indicated that immune protection of MIC3 is influenced by multiple factors. Adjuvants play an important role in the efficacy of immunizations. It is the general consensus of opinion that a type-1 response, particularly associated with CD8+ T cells producing IFN-γ, is the major mediator of immunity against *T. gondii* infection. Other important factors in immunization experiments are the selection of animal model (species/strain) and the selection of *T. gondii* strain. In general, as mice are normally susceptible to *T. gondii* infection, parasite strains with low virulence or low doses of virulent strains may be preferentially selected by some researchers [64]. In some studies of immunization, oral challenges were made with cystogenic strains [65]. It is important to point out that unnatural routes of infection may be key determinants of mouse survival.

### Studies on diagnostic reagents based on MIC3 of *T. gondii*

MIC3 is expressed at all stages of the life cycle of *T. gondii*. Therefore, MIC3 is not only an important candidate molecule for vaccines but also a very promising candidate molecule for the diagnosis of toxoplasmosis. Immunological diagnostic methods based on MIC3 of *T. gondii* include the indirect fluorescent antibody test (IFAT), latex agglutination test (LAT), enzyme-linked immunosorbent assay (ELISA), polymerase chain reaction (PCR), and so on. Jiang *et al.* [66] used recombinant *T. gondii* MIC3 (rMIC3) as an antigen to establish a LAT; there was no cross-reactivity with the standard positive sera of other pathogens, such as classical swine fever virus and foot and mouth disease virus, but there was a strong agglutination reaction with *T. gondii* antibody-positive serum samples from pigs. In addition, compared to the ELISA method using rSAG1 as the antigen, the coincidence rate of sera that tested positive using these two methods was 92.8 %, and this LAT could detect specific *T. gondii* antibodies in all experimental piglets infected with *T. gondii* tachyzoites at 8 to 42 days after infection. Beghetto *et al.* [67] used rMIC3 as an antigen to establish an immunoglobulin G avidity assay, and this assay could detect low-avidity IgG antibodies exclusively in sera collected within 2 months after primary infection. The presence of low-avidity IgG antibodies against rMIC3 antigen can be used to determine the point of infection with *T. gondii* within a 2-month time frame after infection. Furthermore, another research group also demonstrated that particular peptides from MIC3 (MIC3–282:GVEVTLAEKCEKEFGI; MIC3–191:SKRGNAKCGPNGTCIV) were recognized with a significantly higher intensity by sera from acutely infected patients than by sera from latently infected patients, and that these peptides may be candidates for a promising peptide panel for the diagnosis of acute toxoplasmosis in humans [68]. These results indicated that MIC3 is a very good marker for diagnosing recently acquired infections and has potential clinical usefulness for diagnosing the acute phase of *T. gondii* infection during pregnancy. Furthermore, the mic3 gene is also a target for molecular diagnosis of toxoplasmosis. Du *et al.* [69, 70] used the *T. gondii* mic3 gene as a target gene to design specific primers to establish a loop-mediated isothermal amplification (LAMP) assay to detect *T. gondii* in soil. This method had high sensitivity and could detect 5 *T. gondii* oocysts in 0.5 g of soil, providing a rapid, sensitive, and specific molecular detection method for diagnosis of toxoplasmosis.

### Ethical approval

Ethical approval was obtained from the Ethical Committee of the Lanzhou Veterinary Research Institute, Chinese Academy of Agricultural Sciences.

## Conclusions

MIC3 is a very important protein adhesion factor secreted by *T. gondii* that is expressed at the tachyzoite, bradyzoite, and sporozoite stages. It is closely associated with the interaction with host cells and parasites, plays important roles in the early stage of host cell invasion by parasites and is a highly promising candidate factor for vaccine development and diagnosis of toxoplasmosis. Currently, studies on MICs have made great progress, and their structures and functions are becoming clear. The EGF-like domain of MIC3 may have protein adhesion and signal recognition functions during the process of invasion of host cells by *T. gondii*. The correct folding, proteolysis, and expression time of these domains also strictly affect the function of MIC3 in parasites. Therefore, the use of methods such as bioinformatics analysis, fluorescence labeling, establishment of overexpression parasites, gene knockout, gene chips, and high-throughput genome sequencing to study the functions of MIC3 may yield further breakthroughs in parasite-host interaction, signal recognition, and new functions of MIC3.
